# A randomised controlled trial investigating the effect of improving the cleaning and disinfection of shared medical equipment on healthcare-associated infections: the CLEaning and Enhanced disiNfection (CLEEN) study

**DOI:** 10.1186/s13063-023-07144-z

**Published:** 2023-02-22

**Authors:** Katrina Browne, Nicole White, Peta Tehan, Philip L Russo, Maham Amin, Andrew J. Stewardson, Allen C. Cheng, Kirsty Graham, Gabrielle O’Kane, Jennie King, Martin Kiernan, David Brain, Brett G. Mitchell

**Affiliations:** 1 Avondale University, Cooranbong, Australia; 2 Queensland University of Technology, Brisbane, Australia; 3 Monash University, Melbourne, Australia; 4 Cabrini Health, Melbourne, Australia; 5 Central Coast Local Health District, Gosford, Australia; 6 Monash Health, Melbourne, Australia; 7 NSW Health Pathology, Gosford, Australia; 8 University of Newcastle, Newcastle, Australia; 9 University of West London, London, UK

**Keywords:** Hospitals, Cleaning, Cross-infection, Cost-effectiveness, Shared medical equipment, Infection control, Health services, Disinfection

## Abstract

**Background:**

Healthcare-associated infections (HAIs) are a common, costly, yet largely preventable complication impacting patients in healthcare settings globally. Improving routine cleaning and disinfection of the hospital environment has been shown to reduce the risk of HAI. Contaminated shared medical equipment presents a primary transmission route for infectious pathogens, yet is rarely studied. The CLEEN study will assess how enhanced cleaning and disinfection of shared medical equipment affects the rate of HAIs in a tertiary hospital setting. The initiative is an evidence-based approach combining staff training, auditing and feedback to environmental services staff to enhance cleaning and disinfection practices.

**Methods:**

The CLEEN study will use a stepped wedge randomised controlled design in 10 wards of one large Australian hospital over 36 weeks. The intervention will consist of 3 additional hours per weekday for the dedicated cleaning and disinfection of shared medical equipment on each ward. The primary outcome is to demonstrate the effectiveness of improving the quality and frequency of cleaning shared medical equipment in reducing HAIs, as measured by a HAI point prevalence study (PPS). The secondary outcomes include the thoroughness of equipment cleaning assessed using fluorescent marker technology and the cost-effectiveness of the intervention.

**Discussion:**

Evidence from the CLEEN study will contribute to future policy and practice guidelines about the cleaning and disinfection of shared medical equipment. It will be used by healthcare leaders and clinicians to inform decision-making and implementation of best-practice infection prevention strategies to reduce HAIs in healthcare facilities.

**Trial registration:**

Australia New Zealand Clinical Trial Registry ACTRN12622001143718.

## Administrative information

Note: the numbers in curly brackets in this protocol refer to SPIRIT checklist item numbers. The order of the items has been modified to group similar items (see http://www.equator-network.org/reporting-guidelines/spirit-2013-statement-defining-standard-protocol-items-for-clinical-trials/).

**Table:** 

Title {1}	A randomised controlled trial investigating the effect of improving the cleaning and disinfection of shared medical equipment on healthcare-associated infections. The CLEaning and Enhanced disiNfection (CLEEN) study.
Trial registration {2a and 2b}.	The trial is registered with the Australia New Zealand Clinical Trial Registry (ACTRN12622001143718).
Protocol version {3}	V1.1
Funding {4}	This project is funded by an NHMRC Emerging Leadership Investigator grant (Prof Brett Mitchell), (GNT2008392), administered by Avondale University. Academic and clinical project partners at Cabrini Health, Monash University, Alfred Health the Queensland University of Technology and Central Coast Local Health District. Industry partners GAMA Health Care may provide tools to support data collection and education. They have no role in the study design, implementation, analyses, interpretation or publications.
Author details {5a}	**Prof Brett G Mitchell** Avondale University University of Newcastle Monash University Central Coast Local Health District **A/Prof Philip Russo** Cabrini Health Monash University **Dr Nicole White** Queensland University of Technology **Maham Amin** Central Coast Local Health District **Dr Peta Tehan** Avondale University Monash University **Katrina Browne** Avondale University **A/Prof Andrew J Stewardson** Monash University Alfred Health **Prof Allen Cheng** Monash University Alfred Health **Kirsty Graham** Central Coast Local Health District **Dr Gabrielle O’Kane** NSW Health Pathology **Dr Jennie King** Central Coast Local Health District University of Newcastle **Martin Kiernan** University of West London Avondale University **Dr David Brain** Queensland University of Technology
Name and contact information for the trial sponsor {5b}	Avondale University Contact: Prof Brett Mitchell Brett.mitchell@avondale.edu.au
Role of sponsor {5c}	Avondale University staff will be responsible for study design, collection, management, analysis, interpretation of data, writing of reports, journal article submission – Led by the lead investigator Prof. Brett Mitchell.

## Introduction

### Background and rationale {6a}

One in 10 patients in an Australian hospital will acquire an infection while in the hospital (1). This equates to approximately 170,000 infections each year (2). These hospital-acquired infections represent just a subset of the total number of healthcare-associated infections (HAIs), which include infections associated with receiving healthcare in all settings. The burden of HAIs is significant, with increased levels of morbidity and mortality and, for those acquired in hospitals, increased length of stay (2,3). However, there is little level 1 evidence into the role of environmental cleaning to reduce HAIs (4,5). Prevention of infections through practical, implementable and translatable interventions is of critical importance in the era of antimicrobial resistance. These strategies will not only reduce the burden and impact for patients and health services in the short term, but also contribute to limiting antimicrobial resistance and better prepare healthcare settings for emerging infectious disease threats.

Contaminated healthcare environments provide a reservoir for pathogens to be transmitted to patients. Admission to a hospital room previously occupied by a patient infected and/or colonised with a specific pathogen is a major risk factor for acquisition (6). This means that a primary transmission route of pathogens is via the patient environment. A seminal study published in 2020, conducted in 11 Australian hospitals (REACH study), demonstrated improvements in routine hospital cleaning being associated with a reduction in infections (7). This research was the first cluster randomised control trial (RCT) to evaluate the effect of a cleaning and disinfection bundle that focuses on routine and discharge hospital cleaning on the incidence of HAIs. The REACH study did not, however, focus on the cleaning of shared medical equipment. This is a significant lacuna because infectious pathogens have been identified on shared medical equipment (8). Another Australian study published in 2018 demonstrated through whole genome sequencing that shared medical equipment also plays a critical role in the transmission of infection (8). This proposed trial builds on the REACH study findings, including important implementation strategies, and focuses on improving the cleaning and disinfection of shared medical equipment as a key strategy in the reduction of HAIs.

## Objectives {7}

Objective 1: Assess the effectiveness of improving the quality and frequency of cleaning and disinfection of shared medical equipment in reducing HAIs.

Objective 2: Evaluate the cost-effectiveness of the intervention.

## Trial design {8}

**Fig. 1:**
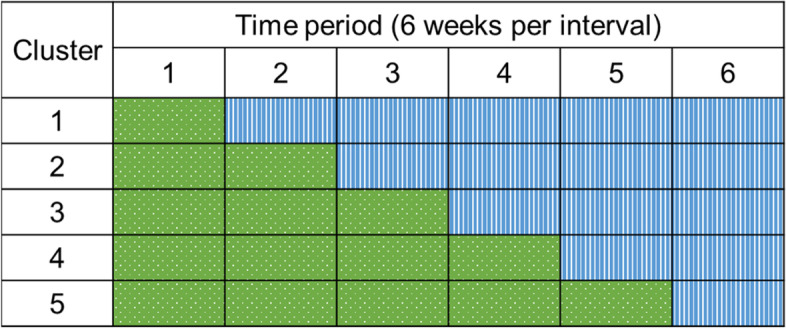
The stepped wedge study design. Green (dotted) = control, blue (striped) = intervention

This study is a stepped wedge randomised controlled study, conducted in 10 wards of one acute Australian hospital over 36 weeks. A stepped wedge design allows all wards to receive the intervention at different times, with each ward acting as its own control. Each cluster will comprise two wards. One cluster will switch to the intervention every 6 weeks. This study design reduces the influences of confounders such as variations in size and case mix and supports feasibility while maintaining rigour. This design also allows research staff to work with individual wards as they adopt the intervention, maximising consistency of intervention and aiding implementation (Fig. 1).

## Methods: participants, interventions and outcomes

### Study setting {9}

One Australian hospital that meets the eligibility criteria will be enrolled in the study. Ten wards within the hospital will be selected for the study.

### Eligibility criteria {10}

The eligible hospital will meet the following criteria: classified as a ‘Public acute group A hospital’ by the Australian Institute of Health and Welfare (9), located in New South Wales, have an intensive care unit, have a minimum of 10 wards, and caring for adult patients (i.e. ≥ 18 years old) willing to participate in the study. Ten wards with > 20 beds, caring for adult patients, will be selected for the study.

Hospitals will be excluded from the study if they do not meet the inclusion criteria. Hospitals may also be excluded from the study if within the study time frame (2023–2024) they are opening, closing or relocating, or implementing major environmental cleaning initiatives or changes.

### Who will take informed consent? {26a}

This project collects data associated with human beings only. Data from all patients (> 18 years old) who are inpatients on one of the 10 wards on the day of the PPS will be used. As this project collects data associated with human beings only, there is no consent process and a waiver of consent has been sought and granted by the authorising Human Research Ethics Committee (HREC). The approach taken in this study with respect to a waiver of consent is consistent with other multi-centre studies where a point prevalence study of healthcare-associated infections has been undertaken as the primary outcome (and data collection method) and ethics approval has been granted (1,10,11). Similarly, our approach is consistent with stepped wedge trials with infections as the outcome and interventions that posed low risk and collected data from existing records. In these instances, a waiver of consent was granted.

There are no interventions and no harm or discomfort to the patient as a result of the project. The benefits of the research justify any risk of harm associated with not obtaining consent. Results of the research are not individualised or patient identifiable. The study requires no direct involvement of patients; rather, it collates existing information obtained during their hospitalisation. No new information will be obtained about individual patients; therefore, results will have no significance for the individual welfare of patients.

### Additional consent provisions for collection and use of participant data and biological specimens {26b}

Not applicable—no biological specimens will be collected for this study.

## Interventions

### Explanation for the choice of comparators {6b}

The stepped wedge study design allows each ward to act as its own control. During the control phase, the cleaning and disinfection of shared medical equipment are undertaken by PSAs according to hospital policies. The intervention phase introduces 15 additional hours per week, purely dedicated to the cleaning and disinfection of shared medical equipment. During the intervention phase, PSAs will be educated on correct cleaning and disinfection technique. To confirm equipment is being properly cleaned during the intervention, auditing with fluorescent markers (FM) will allow feedback to PSA staff. This design allows the comparison between routine (control) and enhanced (intervention) disinfection.

### Intervention description {11a}

The intervention will consist of additional time dedicated to cleaning and disinfection of shared medical equipment, education on cleaning technique, auditing of cleaning efficacy and feedback on cleanliness to staff. This multimodal strategy to improve the cleaning and disinfection of shared patient equipment is grounded in the literature and recent research (7,12). The primary target for the intervention is the cleaning staff.

#### Additional cleaning

During the intervention phase, each trial ward will receive an additional 3 h of cleaning per weekday by dedicated cleaners. This is in addition to routine cleaning that already occurs. The use of dedicated cleaners will reduce the risk of contamination and assist in consistency in implementation. An expression of interest process will be used to identify cleaners who are seeking additional work. This will be managed and handled by the participating hospital, in line with enterprise bargaining arrangements and local policy.

The additional cleaning will focus on specific shared medical equipment (Table 1). The use of a Therapeutic Goods Administration (TGA)-registered dual detergent-disinfectant wipe effective against a range of bacteria and viruses will be used for additional cleaning. The fidelity of the additional cleaning will be measured using the fluorescent markers (see the ‘Audit and feedback’ section). In addition, items that have been cleaned but remain in storage until use will be labelled as cleaned, using bright labelling. This will enable clinical staff to easily identify which equipment is clean and ready for use.

**Table 1:** Focus of additional cleaning

Type of equipment	Additional detail	Frequency	Estimated time
Intravenous drip stand/pole	Those in use by patients	Daily	5 min
	Those in the storage on the ward	Daily	10 min
Infusion pump(s)	Those *not in use* by patients. Areas to be cleaned include all external surfaces daily	Daily	10 min
Mobile blood pressure machines	Automatic machines Areas to be cleaned are cuff, buttons, O_2_ probe, temperature probe, and storage basket	Daily	30 min
Computer on wheels (approx. 10–15 per ward)	Surfaces to be cleaned include: • Horizontal surfaces • Keyboards • Vertical surface and pedestals	Daily Daily Weekly	60 min
Commodes	All surfaces to be cleaned, focus on handles and seat	Daily	20 min
Blood glucose machine	Machines include those in a box and the smaller glucose machines. Focus on cleaning surfaces	Daily	10 min
Mobility equipment	Focus on cleaning frequency touch areas, e.g. handles. Those in storage/shared areas, i.e. not those kept in patient rooms	Daily	20 min
Total			165 min

^Note that if time allows, other equipment will include trolleys, bladder scanners (cleaning probe and buttons), patslides (those in shared areas/storage only), resuscitation trolleys (top surface and drawers—outside only) and medication trolleys

#### Education

The dedicated cleaners undertaking the additional cleaning will undergo education and training sessions prior to the commencement of the intervention. Education prior to the invention will include at least a 1-h in-service, where cleaning techniques will be taught and practised. This will occur no longer than 2 months prior to the commencement of the intervention. The training will be delivered by staff experienced in education and cleaning practice. Standard operating procedures for cleaning specific items of shared medical equipment will be developed and subsequently explained at the in-service (if they are not already in existence at the hospital). These will also be readily available for staff for future reference as needed.

#### Audit and feedback

The benefits and role of audit, including the use of fluorescent markers (FM), in assessing environmental cleaning, is well documented (7,13,14). Audit and feedback to the cleaning staff involved in the additional cleaning will include the use of FM technology, in which invisible gel dots that are removed by cleaning are applied to surfaces. The dots are invisible to the naked eye, resist dry abrasion, and are removed completely by routine cleaning. A structured auditing approach, with staff feedback underpinned by education, will aid improvements in cleaning, as shown in multiple studies (7,13,14). FM auditing of shared equipment will be conducted fortnightly in each ward.

During the intervention phase, audit results will be reported verbally to the cleaning staff, with a follow-up email to cleaning supervisors and the Nursing Unit Managers. Audit results will be used to inform further informal face-to-face education and training. In discussing audit results, informal goals for the next audit will be set with the cleaners. Additional reports will be provided for internal committees, such as the Infection Prevention and Control committee at the conclusion of the study.

### Criteria for discontinuing or modifying allocated interventions {11b}

The study will be discontinued if a regulatory body, funding body or the HREC judges it necessary for medical, safety, regulatory or other reasons consistent with applicable laws, regulations and good clinical practice.

### Strategies to improve adherence to interventions {11c}

To improve adherence to intervention protocols, there will be educational training sessions that focus on correct cleaning techniques. Refresher training sessions for PSAs will be held during the intervention period to promote correct cleaning and disinfection techniques. The study coordinator will have regular communication with PSAs (fortnightly) where the results of the FM audits will be communicated to PSA staff to help improve the quality of cleaning and disinfection. During the intervention phase, PSAs allocated to additional cleaning hours will have daily communication with Nursing Unit Managers.

### Relevant concomitant care permitted or prohibited during the trial {11d}

Not applicable—as there is no direct intervention for patients.

### Provisions for post-trial care {30}

Not applicable—as there is no intervention directly to participants. The intervention, additional hospital cleaning, carries no additional risk to participants.

### Outcomes {12}

The primary outcome is the proportion of inpatients aged ≥ 18 years old with a HAI as measured by a HAI point prevalence study (PPS). The secondary outcomes are the thoroughness of cleaning as being the proportion of dots that were completely removed, as measured by the FM gel and ultraviolet light system, and the cost-effectiveness of the intervention compared with routine cleaning.

### *Participant timeline{13}*

**Fig. 2:**
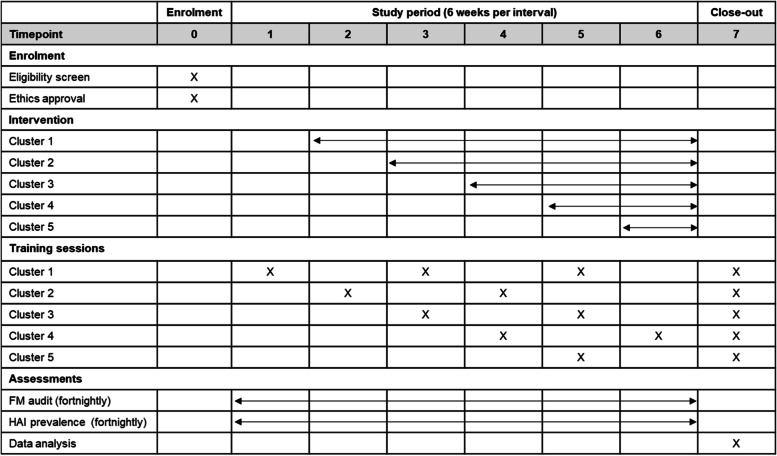
Timeline for enrolment, interventions and assessments of the study. FM fluorescent markers, HAI healthcare-associated infection

The participant timeline is shown in Fig. 2.

### Sample size {14}

The study design ensures a sample size sufficient to detect a relative reduction of at least 35% in total HAI infection, with a baseline point prevalence of 11%. It is powered at 80%, based on a two-sided 5% significance level with an inter-cluster correlation of 0.3 and coefficient of variation of 0.65—allowing for variation in cluster size. A stepped wedge sample size formula was used, considering the number and size of clusters and the time period. The required cluster size is 132 (66 per ward), achieved with two wards forming clusters and three fortnightly PPS each time period (*n* = 3960—5 clusters, 6 time periods). The baseline infection rate was determined using recently published work by the applicant and reductions identified in a recent RCT (3,15). Varying the intra-cluster correlation between 0.2 and 0.5 produced minimum sample sizes per cluster period of 94 (47 per ward) to 150 (75 per ward).

### Recruitment {15}

In alignment with past approaches in Australia and the European Centre for Disease Prevention and Control protocol, all patients admitted to the ward before or at 08:00 h on the first survey day and not discharged from the ward at the time of the survey will be eligible and will be evaluated for a HAI. Patients transferred in or out after 08:00 h will not be included. Patients will be excluded if they are under 18 years of age or are due to have same-day treatment or surgery.

## Assignment of interventions: allocation

### Sequence generation {16a}

There will be two wards allocated to each cluster. The allocation of when each cluster will commence the intervention will be randomised using Microsoft Excel. This will be undertaken by the study statistician, not involved in data collection or the determination of outcomes. The equipment to be tested in the FM audit will be randomised using Microsoft Excel to ensure a variety of shared medical equipment is audited. This will be undertaken by one member of the research team, not involved in data collection or the determination of outcomes.

### Concealment mechanism {16b}

Once randomisation has occurred and each ward is allocated into a cluster, this information will be kept by the person undertaking randomisation on their own (work) computer. This allocation will not be available to the participating wards or PSAs or those involved in data collection. Each participating ward and PSAs will be informed of the cluster allocation 2 weeks prior to the commencement of the intervention of that cluster. This concealment process will be undertaken by the person undertaking the randomisation informing the trial coordinator of the allocation, as per the timing above. The trial coordinator will then inform the ward and PSAs.

### Implementation {16c}

The allocation sequence will be generated by the study statistician and each ward will be randomly assigned to a cluster. As each cluster enters the intervention phase, the trial coordinator will facilitate PSA training sessions and auditing feedback.

## Assignment of interventions: blinding

### Who will be blinded {17a}

The researcher collecting the data on the primary outcome (HAI data) will be blinded to the intervention (i.e. knowing whether the ward is an intervention or control ward). Similarly, the researcher collecting FM audit data will be blinded to the intervention. Results of the audit process will not be made available to wards in the control phase of the study.

### Procedure for unblinding if needed {17b}

Once data collection and statistical analysis have been finalised, the interventions can be unblinded for the purpose of interpreting the data.

## Data collection and management

### Plans for assessment and collection of outcomes {18a}

A data extraction tool has been developed and piloted in REDCap®. This tool was adapted from a previous multi-centre point prevalence study (1). The researcher responsible for collecting the data will be trained in the use of the survey instrument to extract relevant data pertaining to the intervention and relevant outcomes. After reviewing medical records, pathology and microbiology databases, the researcher will determine if the patient has a HAI. The determination of a HAI will be undertaken through an algorithm applying the HAI definitions in the ECDC protocol (16). Data on each HAI identified will be consistent with the ECDC protocol. The attribution of a HAI to a ward will be determined through the use of a 48-h time frame, i.e. the infection symptom onset must occur > 48 h after admission to the ward, for it to be attributable to the ward. If a patient is transferred to a ward and a HAI is identified within 48 h of transfer, it will be attributed to the previous ward. An interrater reliability assessment will be independently undertaken to validate the accuracy of HAI determinations (1).

### Plans to promote participant retention and complete follow-up {18b}

Not applicable—as patients are not recruited and there is no patient follow-up in this study.

### Data management {19}

The data which will be obtained from patients’ hospital medical record will be non-identifiable and there are clear processes in place to ensure that the privacy of data is maintained. These processes include appropriate storage of data in password-protected files and the destruction of these data after completion of the study. The data will be kept for a period of 5 years from the point of any publication relating to the research.

### Confidentiality {27}

No identifiable data is collected. The project is supervised and overseen by a clinician, registered with the Australian Health Practitioner Regulation Authority.

### Plans for collection, laboratory evaluation and storage of biological specimens for genetic or molecular analysis in this trial/future use {33}

There will be no biological specimens tested in this study.

## Statistical methods

### Statistical methods for primary and secondary outcomes {20a}

#### Primary outcome analysis

The primary outcome is defined as the total fortnightly rates of HAI identified. The primary outcome will be analysed by a generalised linear mixed model (GLMM) with a logit link function, where the dependent variable is defined as will be used, to estimate changes in fortnightly cases of HAI. To standardise rates, the fortnightly numbers of HAIs identified (combined, all types of HAIs) will be divided by the number of at-risk patients. Models will have a random intercept for each ward to control for baseline differences. Fixed effects will include the intervention and study time in weeks and fixed effects for the intervention and study time. Study time will be modelled as a categorical fixed effect; inclusion as a linear fixed effect will also be tested. Models will have a random intercept for each ward to control for baseline differences between wards, and a linear fixed effect to control for unrelated changes over time. Intervention effectiveness will be represented in the model as a binary independent variable that will for the intervention switched from ‘no’ (0) to ‘yes’ (1) 1 week after the start of the intervention period. This will account for a delay in the intervention effect, as wards become familiarised with the enhanced cleaning procedures. Model estimates will be reported as odds ratios with 95% confidence.

We will undertake sensitivity analyses to determine the possibility of a delayed intervention effect of longer than 1 week, the influence of each ward on model estimates and the effect of the intervention on the most common and serious HAIs—pneumonia, urinary tract infections, bloodstream infections and surgical site infection. The delayed intervention effect will be modelled at 2 and 4 weeks after each ward’s intervention start date. The influence of each ward will be examined using a leave-one-ward-out analysis examining changes to the intervention effect and Cook’s distances.

Additional analyses will consider alternative model specifications if the proposed GLMM does not converge or provides an inadequate fit to the data. Planned alternatives will be testing of different link functions (identity, log), changing the distribution for the dependent variable from binomial to beta-binomial to account for over-dispersion, and the use of generalised estimating equations in place of a GLMM.

To reduce the effect of confounders due to seasonality, viral respiratory infections (RSV, COVID-19, influenza) will be removed for the purpose of primary analysis. In addition, any HAI data related to an outbreak will be excluded from primary analysis. The study period is aligned to span over three full seasons (autumn, winter, spring), with the study midpoint corresponding to the usual epidemiological peak for respiratory infection in the region. Additionally, the use of historical coding data can identify underlying trends over recent years.

#### Secondary outcome analysis

##### FM audits

We will analyse data from fortnightly cleaning audits using a binomial generalised linear mixed model with a logit link function on the proportion of equipment that were deemed ‘cleaned’. A random intercept will be included for each ward. The effect of the intervention will be tested in three ways: a binary intervention effect, to model an immediate improvement in cleaning; a linear intervention effect, defined as weeks after each ward’s intervention start date, to model a more gradual improvement over time; and a combined binary–linear intervention effect. As per the analysis of the primary outcome, the intervention effect will switch from ‘no’ to ‘yes’ 1 week after the start of the intervention period.

##### Cost-effectiveness

The costs of adopting the intervention will be prospectively collected from hospital records, such as the cost of cleaning consumables. Excess length-of-stay estimates will be sourced from studies identified by systematic reviews to estimate the value of bed days saved. The cost-effectiveness of the intervention will be evaluated from the perspective of the hospital decision-maker. Cost-effectiveness will be summarised by the incremental cost-effectiveness ratio and net monetary benefit, which offer different summaries of the change in costs versus health benefits. Modelled changes in the primary outcome (HAIs) will be used to estimate changes in health benefits from cases prevented by the intervention, as quality-adjusted life year (QALY). Probabilistic sensitivity analysis will be undertaken to account for uncertainty in model parameters and its impact on cost-effectiveness outcomes.

Uncertainties in parameter estimates will be captured using appropriate statistical distributions to describe the variability. The fitted distributions will be subject to random re-samples simulated 10,000 times. The distributions of all prior parameters are used to estimate the posterior distributions of ‘change to costs’ and ‘change to QALY’ outcomes. The decision will be informed by plotting cost-effectiveness acceptability curves with threshold values between zero and 100,000 per QALY gained and using the net monetary benefits framework. These approaches are semi-Bayesian and appropriately account for all parameter uncertainty for the adoption decisions.

### Interim analyses {21b}

There will be no interim analysis as we do not expect there to be any negative effects from this study.

### Methods for additional analyses (e.g. subgroup analyses) {20b}

Not applicable—there are no subgroup analyses planned.

### Methods in analysis to handle protocol non-adherence and any statistical methods to handle missing data {20c}

Statistical analyses will be performed as intention-to-treat, in accordance with the published protocol. For the specified study design, the intention-to-treat analysis will assume cluster transitions from control to intervention according to randomised sequence allocation and include all patients who meet the study inclusion criteria.

Missing data will be handled by complete case analysis. Given the use of standardised data collection instruments and protocols, missing data on primary and secondary outcomes is expected to be low. Any deviations from the protocol will be documented and reported in planned research outputs.

### Plans to give access to the full protocol, participant-level data and statistical code {31c}

Fully de-identified data sets and statistical codes will only be available by contacting a chief investigator and providing the appropriate ethical approvals.

## Oversight and monitoring

### Composition of the coordinating centre and trial steering committee {5d}

The project consists of a management and steering committee. The management committee (coordinating centre) will oversee the day-to-day running of the trial and decide on the operational elements of the trial. Members of the coordinating centre are two chief investigators and the trial coordinator. The steering committee will meet as required to provide oversight of the study. The steering committee will have accountability and responsibility for the project, including progress towards completion of agreed project activities (milestones/ deliverables), risks arising and how these are being managed to ensure project outcomes, and research reports. The steering committee consists of all chief investigators and the trial coordinator. Associate investigators are non-voting members of the Steering Committee.

### Composition of the data monitoring committee, its role and reporting structure {21a}

The study does not have a data monitoring committee. The intervention is an enhancement of existing health services delivery (cleaning) with no anticipated risks. There is no planned interim analysis.

### Adverse event reporting and harms {22}

The trial coordinator is responsible for ensuring that all adverse events observed by the investigator/s, study team or reported by sites are collected and recorded in the source documents. The trial coordinator will notify the approving ethics committee of serious adverse events occurring at any of the sites. Adverse events could require reporting as per hospital-specific policy.

### Frequency and plans for auditing trial conduct {23}

The HREC granting approval for this study may conduct an external audit at any point in the trial. The steering committee will be responsible for monitoring risks and the progress of the trial.

### Plans for communicating important protocol amendments to relevant parties (e.g. trial participants, ethical committees) {25}

Where there are any ethical amendments to the trial protocol, approval will be sought from the approving HREC. Similarly, important protocol modification will be updated on the Australian and New Zealand Clinical Trial registry.

## Dissemination plans {31a}

The investigators will implement a dissemination plan that will include key communication strategies for all stakeholders, an open-access publication plan, authorship requirements and publication standards that align with NHMRC Australian Code for the Responsible Conduct of Research (http://www.nhmrc.gov.au/guidelines-publications/r39) and an International Committee of Medical Journal Editors Recommendations for the Conduct, Reporting, Editing, and Publication of Scholarly Work in Medical Journals (http://www.icmje.org/recommendations/browse/roles-and-responsibilities/defining-the-role-of-authors-and-contributors.html).

## Discussion

The CLEEN study is a large cluster randomised controlled trial conducted in an Australian tertiary hospital. The study represents the first randomised controlled study in the world to provide level 1 evidence on the impact of additional cleaning of shared equipment on rates of HAIs (5). The advantages of the study design include a simple and feasible intervention, with strategies built in to ensure adherence and fidelity. Unlike pragmatic trial designs, which may use existing resources of PSAs, we are able to ensure that the intervention is delivered through additional resources being provided specifically for the purpose of delivering the intervention. The stepped wedge design is another strength of the research design, as each ward will act as its own control. This negates issues associated with differing patient case mix, individual ward culture, operational issues and local practices. This study will provide evidence to inform the development of a future service delivery model, national guidelines and local policy with respect to roles and responsibilities and the importance of cleaning of shared medical equipment.

Limitations to the study may include issues such as hospital changes in policy or practice that may influence the outcomes being observed. However, the hospital is committed to ensuring that there will be no significant changes to the hospital cleaning policy during the trial period unless absolutely necessary. Other aspects of infection prevention control policy such as hand hygiene and antimicrobial usage will be monitored, and we will be able to document/identify changes in other policies that may influence the outcomes of this study. Further potential disadvantages of the trial may include that the intervention will only be delivered 5 days of 7, due to physical and financial constraints. Given the intervention is being delivered over the vast majority of the working week, the burden and risk in the environment will still likely be significantly reduced. Environmental swabbing of the environment was considered; however, due to poor positive predictive value, it was not included in the current study design (17). In a related study in the same participating hospital, we intend to use targeted whole genome sequencing to evaluate potential transmission pathways through shared medical equipment.

## Trial status

The trial protocol V1.1. is the current and approved version as of 21 September 2022. Hospital recruitment is completed, including all required ethical approvals. Data collection will begin in March 2023 with an expected end date for data collection in November 2023.

## Data Availability

Details about the study will be posted on information boards in the participating hospital. Standard operating procedures for cleaning specific items of shared medical equipment will be developed and subsequently explained at the in-service (if they are not already in existence at the hospital). These will also be readily available for staff for future reference as needed. Availability of data may be available upon reasonable request, by contacting a chief investigator and providing the appropriate ethical approvals.

## Abbreviations

HAI: Healthcare-associated infection
CLEEN: CLEaning and Enhanced disiNfection
NHMRC: National Health and Medical Research Council
RCT: Randomised control trial
REACH: Researching Effective Approaches to Cleaning in Hospitals
TGA: Therapeutic Goods Administration
FM: Fluorescent markers
HREC: Human Research Ethics Committee
PPS: Point prevalence survey
QALY: Quality-adjusted life year
